# HPV16 E6/E7 -based mRNA vaccine is therapeutic in mice bearing aggressive HPV-positive lesions

**DOI:** 10.3389/fimmu.2023.1213285

**Published:** 2023-07-12

**Authors:** Kun Zhou, Olga Yuzhakov, Nouredine Behloul, Dehua Wang, Lakshmi Bhagat, Dafeng Chu, Xinyue Zhang, Xinwei Cheng, Lusheng Fan, Xinyu Huang, Teodelinda Mirabella

**Affiliations:** ^1^ R&D Department, GeneLeap Biotechnology, Woburn, MA, United States; ^2^ R&D Department, Nanjing GeneLeap Biotechnology, Nanjing, China

**Keywords:** mRNA, lipid nanoparticles, HPV, therapeutic vaccine, tumors

## Abstract

HPV (Human papillomavirus) affects 600,000 people worldwide each year. Almost all cervical cancers are associated with a past HPV infection. In particular, the positivity to the high-risk type HPV16 is detected in most of the invasive cervical cancers. FDA has approved prophylactic vaccines that protect against new HPV16 infections, but do not induce immunity in those patients with established infections or neoplasms. To date, no therapeutic vaccine targeting HPV16-associated lesions has been authorized. We have developed an mRNA-based vaccine against the HPV16 late oncoproteins E6 and E7, which are abundantly and exclusively expressed in high-grade squamous intraepithelial lesions (HSILs), a stage of the cervical disease that precedes the progression to carcinoma. Our *in vitro* and *in vivo* studies demonstrated that the translated mRNA is functional and elicits an antigen-specific adaptive immune response. Upon immunization with the vaccine, mice with HPV16+ lesions exhibited tumor growth inhibition, extension of lifespan, and development of a protective immune memory. In light of these results and the remarkable clinical success of mRNA vaccines against SARS-CoV2, we believe that our mRNA-based therapeutic vaccine has the potential to offer a non-invasive treatment alternative to the current standard of care for HPV16+ HSILs.

## 1 Introduction

At some point in life, most sexually active women and men become infected with Human papillomavirus (HPV), a small non-enveloped circular double-stranded DNA virus from the Papillomaviridae family (1). There are over 200 different genotypes of HPVs, which are further subdivided into low-risk and high-risk types based on their association with cancer or precancerous lesions (1, 2). Over 99% of carcinomas of the cervix are associated with HPV infection. In particular, the positivity to the high-risk type HPV16 is detected in more than 60% of invasive cervical cancers (3, 4).

Typically, HPV16 causes a transient infection that spontaneously regresses within a few months. However, when the immune system fails to clear the infection, the persistence of the virus may cause cellular changes leading to cervical intraepithelial neoplasia (CIN) and cancerous lesions (1, 5). The stages of the cervical disease, CIN1 (or Low-grade squamous intraepithelial lesion, LSIL) and CIN2/CIN3 (or High-grade squamous intraepithelial lesion, HSIL), are traditionally diagnosed with a HPV DNA test to identify the virus genotype, as well as pathological analysis of Pap smears and colposcopy-biopsied tissues to further assess abnormalities in keratinocytes differentiation, thickness and organization of the basal layer (6). The HPV16 episomal DNA genome consists of a 7.9 kb long nucleotide belt, segmented into three regions: the early (E) region encoding for viral proteins regulating the virus life cycle and the cellular functions of the infected epithelial cells, the late (L) region encoding for the structural proteins of the viral icosahedral capsid that mediates cell entry, and the LCR (long control region) containing the cis-acting sequences that mediate viral replication and transcription (1, 7). Prophylactic vaccines, such as Gardasil^®^ and Cervarix^®^, stimulate formation of neutralizing antibodies against the capsid protein L1, preventing virus entry and infection transmission (8). The oncogenic early proteins E6 and E7 are the most targeted antigens by the HPV16 therapeutic vaccines currently under development for several reasons (9): 1) E6 and E7 are abundantly and exclusively expressed in precancerous and cancerous lesions, hence the risk of targeting healthy tissues is not significant; 2) E6 and E7 are essential for transforming and maintaining infected cells transformation, hence the risk of antigen loss-mediated immune escape is not significant; 3) no mechanisms of central tolerance towards E6 and E7 have been documented; 4) the immune response against E6 and E7 has been characterized preclinically and clinically (10). To date, there is no therapeutic HPV vaccine approved by the US FDA, yet. The recent success of mRNA-based COVID vaccines Spikevax^®^ and Comirnaty^®^ in controlling the pandemic demonstrates the safety and potency of this unconventional approach, as well as its adaptability for addressing future public health emergencies (11, 12).

Here, we introduce an mRNA-based vaccine that encodes for modified versions of HPV16 early proteins E6 and E7, is formulated in MC3-based lipid nanoparticle (LNP) and can be administered *via* intramuscular injection. We show that: 1) the mRNA is translated into the desired fusion protein, 2) an antigen-specific adaptive immune response is elicited in vaccinated mice and in a human-relevant co-culture platform, 3) tumor growth is inhibited and survival probability is increased in mice bearing HPV-transformed C3.43 tumors and immunized with the vaccine in therapeutic mode, 4) protective anti-tumor memory is developed in the vaccinated mice that are complete responders. In light of these data, we believe our mRNA vaccine shows great preclinical promise for targeting HPV16-associated HSILs.

## 2 Materials and methods

### 2.1 RNA synthesis and formulation

HPV16 mRNA incorporates HPV16 E6 and E7 sequences NC_001526.4 (7125…7601) and NC_001526.4 (7604…7900) modified according to previously published literature (13). These nucleotide sequences were codon-optimized *via* ATUM Bio algorithm. The DNA2.0 plasmid template (**Supplementary Figure 1**) used for *in vitro* transcription (IVT) of the HPV16 vaccine mRNA was ordered from ATUM Bio, Newark, CA. A T7 promoter, 124A Poly(A) tail and published 5’ UTR and 3’ UTR (14) were incorporated in the plasmid design. Other mRNA sequences used in this article are HPV16 E1, E2 and E5 mRNAs, which incorporate ATUM codon-optimized HPV16 E1 (NC_001526.4 (1.1950)), E2 (NC_001526.4 (1892.2989)), E5 (NC_001526.4 (2986.3237)), respectively, provided with an IgE signal peptide and a FLAG tag to verify expression. The N1-methylpseudouridine (N1mΨ)-modified mRNA was mostly ordered from TriLink Biotech, San Diego, CA. For the *in-vitro* experiments and for the CD4/CD8 profiling of the tumor microenvironment, the mRNA was synthesized in-house *via* a T7-mediated IVT and purified by affinity chromatography to meet the criteria of < 0.1% dsRNA impurities in the final product mass. Briefly, the mRNA was synthetized using HiScribe^®^ T7 High Yield RNA Synthesis Kit (E2040S, NEB) from a BspQI-linearized plasmid DNA template according to the manufacturer’s instructions. The mRNA was co-transcriptionally capped with CleanCap-AG (N-7113, TriLink) incorporated at a final concentration of 4mM. IVT mRNA was also chemically modified by complete substitution of uridine with N1mΨ (N-1081, TriLink). The final mRNA was purified by oligo-dT affinity chromatography (A47384, Thermo Fisher Scientific). The mRNA was formulated in MessengerMax™ for the *in vitro* experiments (see below), and in lipid nanoparticles (LNP) for all the *in vivo* experiments. For the LNP preparation, Dlin-MC3-DMA (Organix Inc, Woburn, MA, USA), DSPC, Cholesterol, and DMG-PEG2000 (all from Avanti Polar Lipids, Alabaster, AL, USA) were dissolved in ethanol at the molar ratio 50:10:38.5:1.5. The mRNA solutions at concentration 0.45 mg/mL (in 20 mM Malic Acid, pH 3) and the lipid solutions at concentration 23.4 mg/mL were mixed in a NanoAssembler Ignite (Precision Nanosystems, Vancouver, BC, Canada), using a flow rate of 12 mL/min and with an aqueous/ethanol volume ratio of 3/1. Formulations were dialyzed overnight against PBS (pH 7.4) with 8% sucrose in Slide-A-Lyzer™ G2 Dialysis Cassettes, 20K MWCO (Thermo Fisher Scientific). Fresh LNP-formulated mRNA was diluted in 5 mM NaCl solution and particle size and PDI were measured in a Zetasizer machine (Malvern Panalytical Ltd, Malvern, UK). The encapsulation efficiency (EE) was measured by the Quant-it ™ Ribogreen Assay Kit (Thermo Fischer Scientific) and an EE threshold was set at ≥95%, unless otherwise indicated. The LNP-formulated mRNAs with particle size of 70-80 nm and PDI ≤ 0.25 were then stored at -70°C until use.

### 2.2 Cell culture and treatments

HEK293T and HeLa cells were obtained from the Chinese National Collection of Authenticated Cell Cultures. THP-1 cells were purchased from the American Type Culture Collection (ATCC). The C3.43 cell line was licensed from Martin Kast at the USC Norris Comprehensive Cancer Center, Los Angeles, CA. HEK293T and HeLa cells were maintained in DMEM supplemented with 10% fetal bovine serum (FBS) and 1% penicillin-streptomycin. THP-1 and human antigen-specific T cells (Charles River Laboratories, #ASTC-1099) were kept in RPMI 1640 supplemented with 1% penicillin-streptomycin, 2mM L-glutamine and 10% FBS. C3.43 cells were cultured in IMDM medium supplemented with 10% FBS, 2 mM L-Glutamine, 1% penicillin-streptomycin and 50μM 2-mercaptoethanol. All the cell lines were tested for mycoplasma negativity and were cultured at 37°C in incubators supplied with 5% CO_2_.

In the experiments requiring mRNA transfection and protein analysis, HeLa and HEK293T cells were seeded in 24-well plates at 150,000 cells/well for an overnight. Cells were transfected with mRNA formulated in Lipofectamine MessengerMax™ Transfection Reagent (LMRNA015, Invitrogen) according to the manufacturer’s instructions. At the desired time-point post-transfection, the supernatant was discarded, and the cells were lysed with RIPA lysis buffer.

For the inhibition of glycosylation, HeLa cells were first treated with 0.25, 0.5 or 1 μg/mL of tunicamycin (HY-A0098/CS-5779, MCE, China) for 2h and then transfected with 500 ng/well of mRNA; the lysates were prepared at 24h post-transfection. For the de-glycosylation assay, the lysate prepared from mRNA-transfected HeLa cells were first denatured at 100°C for 10min, chilled on ice, and then treated with 500 or 250U (per 10 μg of total protein) of Peptide-N-Glycosidase F (PNGase F, P0704S, New England Biolabs) for 1h at 37°C.

### 2.3 Western blotting

The total protein amount in the cell lysates prepared from mRNA-transfected cells was determined using Pierce™ BCA Protein Assay Kit (Thermo Fisher Scientific) according to the manufacturer’s instructions. Next, samples with 10 μg total protein were prepared with SDS loading buffer and β- Mercaptoethanol, denatured at 100°C for 5min, separated by 12% SDS-PAGE gel and transferred to a PVDF using Trans-Blot Turbo Transfer System (Bio-Rad). The membranes were blocked with 5% skimmed milk for 2h at room temperature and then incubated with the primary antibody at a final concentration of 1 μg/mL for 18h at 4°C, and with a HRP-conjugated secondary antibody for 2h at room temperature. Signal was detected using SuperSignal™ West Pico PLUS chemiluminescence enhancer (Thermo Fisher Scientific) and visualized by ChemiDoc MP Imaging System (Bio-Rad). Band intensity was analyzed using GelAnalyzer 19.1.1. HPV16 E7 monoclonal antibody (NM2 clone, sc-65711, Santa Cruz) or HPV16 E6 Polyclonal Antibody (PA5-117355, Invitrogen) were used as primary antibodies with HRP goat anti-mouse lgG H&L (ab6789, Abcam) or HRP goat anti-rabbit lgG H&L (1706515, Bio-Rad) as the corresponding secondary antibodies, respectively. Beta-actin was detected as a housekeeping protein using HRP-beta actin monoclonal antibody (2D4H5 clone, HRP-66009, Proteintech).

### 2.4 THP-1/T cell coculture platform and IFNγ ELISPOT assay

About 4x10^6^ THP-1 cells were seeded in T75 flasks and differentiated towards immature dendritic cells (iDC) over a 5-day culture in 20 mL serum-supplemented RPMI 1640 in the presence of 100 ng/mL hIL4 (R&D Systems, 204-IL-020/CF) and 100 ng/mL hGM-CSF (Sigma-Aldrich, #GF304). To allow full maturation towards mature dendritic cells (mDC), iDC were collected *via* centrifugation, resuspended in serum free RPMI 1640 media supplemented with 200ng/mL hIL4, 100ng/mL hGM-CSF, 20ng/mL hTNFα (Sigma-Aldrich, #GF314), and 200ng/mL ionomycin (Tocris Bioscience, 2092/1), and plated at density 10,000/well in the 96-well plates provided with the ELISPOT assay kit R&D Systems, #EL485, Minneapolis, MN. Cells were kept in culture for 1 day to allow differentiation towards mDC, before beginning the transfection with MessengerMax-formulated mRNA to induce expression of the E6-E7 fusion protein in the vaccine. The day following the transfection, human antigen specific E7_11-20_ T cells (Charles River Laboratories, #ASTC-1099) were added to the 96-well plate at density 20,000 cells/well. ASTC and mDC cells were kept in coculture for an overnight. The plates were processed as per manufacturer (R&D Systems, #EL485)’s instructions and read under the CTL Immunospot Analyzer (ImmunoSpot^®^). CD209 monoclonal antibody eB-h209 (eBioscience™) was used to characterize DC differentiation *via* Flow Cytometry.

### 2.5 Mouse splenocytes isolation and IFNγ ELISPOT assay

Mouse spleens were collected in 50 mL Falcon tubes decked with 70 μm nylon cell strainers and kept in ice. Spleens were gently passed through the cell strainer with the help of syringe plungers. Cells were then centrifuged at 700 g for 5 min at 4 °C. Cell pellets were resuspended with 1 ml Gibco™ ACK RBC Lysis buffer per spleen and incubated for 5 min at room temperature with occasional shaking. The reaction was then stopped by adding 30 ml of RPMI 1640 media supplemented with 10% FBS, 1% penicillin-streptomycin and 50μM beta-mercaptoethanol. After centrifuging at 700 g for 5 min at 4 °C, the cell pellets were resuspended in 10 ml of ice-cold RPMI 1640 media supplemented with 10% FBS, 1% penicillin-streptomycin and 50μM beta-mercaptoethanol, and plated at density of 250,000 cell/well in the 96-well plates provided with the ELISPOT assay kit R&D Systems, #EL485, Minneapolis, MN. Customized E6 or E7 peptide libraries (GenScript) were added to the wells at the final concentration of 5 μg/ml, unless otherwise indicated. After an overnight incubation at 37°C, plates were processed as per manufacturer (R&D Systems, #EL485)’s instructions. All microplates were read under the CTL Immunospot Analyzer (ImmunoSpot^®^).

### 2.6 Animal experiments

C57BL/6 mice were used for both immunogenicity and C3.43 syngeneic tumor studies. Female mice were ordered from Charles River Laboratories (Willington, MA) at 6 to 8 weeks, with a body weight ranging from 18 to 25g at the time of their enrollment in the study.

For the immunogenicity study, 10 to 20 μg of LNP-formulated mRNA doses were administered in a volume of 50 μl, either intramuscularly or subcutaneously. At the end of the study, spleens were collected to test the IFNγ response, either *via* ELISPOT (see above) or *via* intracellular staining with flow cytometry detection. The antibodies used for the flow cytometry analysis were: PE anti-mouse IFNγ Antibody (Biolegend, #505808), CD4 Monoclonal Antibody (RM4-5) Super Bright™ 436 (Invitrogen, #62-0042-82), CD8a Monoclonal Antibody (53-6.7)_Alexa Fluor™ 700 (Invitrogen, #56-0081-82), CD90.2 (Thy-1.2) Monoclonal Antibody (53-2.1)_ Super Bright™ 645 (Invitrogen, #64-0902-82).

For the syngeneic tumor model, a volume of 100μl of cell suspension containing 2x10^6^ C3.43 tumor cells in HBSS was implanted subcutaneously (SC) in the right flank just proximal from the hip. When tumor masses reached ~100 mm^3^, mice were randomized and assigned to different treatments. HPV vaccine mRNA, control mRNA and vehicles were given intramuscularly in the quadricep muscle at a volume of 50 μl. Tumors size was periodically measured with a caliper, and volumes were calculated using the formula 0.5 x Length x Width^2^. For the *in vivo* rechallenge study, the HPV16 mRNA vaccinated animals deemed to be complete responders by Day 59, were enrolled in a subsequent study and matched with 10 naïve C57BL/6 mice. The contralateral flank was SC injected with 2 x 10^6^ C3.43 tumor cells and the tumor size was monitored until day 91.

To calculate the CD4/CD8 ratio within the tumor microenvironment, another study was initiated where tumor masses with a volume not inferior to 120 mm^3^ were explanted and dissociated using the tumor dissociation kit (Miltenyi Biotech, #130-096-730) according to the manufacturer’s instructions. The tumor cells were stained with anti-CD4-BB700 (BD Biosciences, #566407), anti-CD3-APC-Fire750 (Biolegend, #100248), anti-CD8a-BV711 (BioLegend, #100759), anti-CD45-APC (BioLegend, #103112).

All the *in vivo* studies were carried out at Biocytogen, Wakefield, MA, in accordance with the internal Institutional Animal Care and Use Committee (IACUC).

### 2.7 Statistical analyses

Statistical analyses are indicated in Figure legends and were performed using GraphPad Prism 9, with p-values < 0.05 deemed as significant. Data are presented as mean ± SEM (standard error of the mean), unless otherwise indicated.

## 3 Results

### 3.1 HPV16 mRNA vaccine is translated into E6-E7 fusion protein

**Figure 1:**
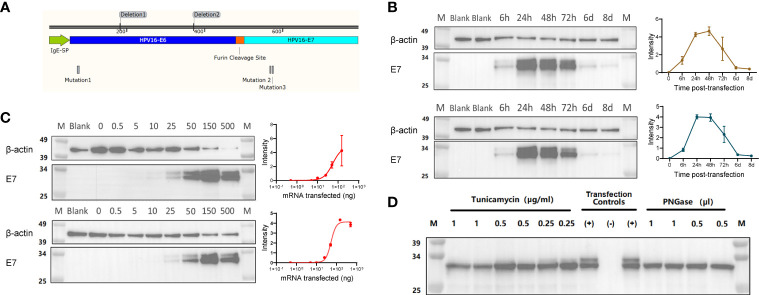
**(A)** Illustration of the fusion protein containing an IgE signal peptide (SP), a modified E6 (non-oncogenic version) linked to a modified E7 (non-oncogenic version) *via* a Furin cleavage site. **(B)** Representative Western Blot assay detecting the HPV16 E6-E7 fusion protein (~31 kDa) with either an Anti-E6 or an Anti-E7 antibody on lysates from HEK293T cells transfected with 0.5 μg mRNA, and analyzed at 6, 24, 48, 72 hours, 6 and 8 days post-transfection. Quantification of band intensity at the indicated time points is shown on the right. **(C)** Representative Western Blot assay detecting the HPV16 E6-E7 fusion protein (~31 kDa) with either an Anti-E6 or an Anti-E7 antibody on lysates from HEK293T cells transfected with different amounts of mRNA (0, 0.5, 10, 25, 50, 150 and 500 ng/well), and analyzed at 24 hours post-transfection. Quantification of band intensity at the indicated time points is shown on the right. **(D)** Western Blot assay detecting the non-glycosylated HPV16 E6-E7 fusion protein (~31 kDa) in lysates of mRNA-transfected HeLa cells using Anti-HPV E7 antibody. The non-glycosylated HPV16 E6-E7 fusion protein was obtained by the inhibition of *in-situ* glycosylation with different concentration of Tunicamycin (μg/mL) or by protein de-glycosylation with PNGase (500U/μl) treatment of the cell lysates. Transfection controls: (+) untreated lysate from mRNA-transfected cells;(-) lysate from non-transfected cells.

The oncoproteins E6 and E7 are well known targets for vaccination strategies against HPV16. As previously described (13), E6 and E7 can be engineered to contain mutations and deletions to prevent their binding to p53 and pRb, respectively, a strategy that is meant to eliminate their oncogenic potential. The mRNA in our HPV16 vaccine encodes a highly efficient IgE leader sequence to facilitate the expression of a fusion protein containing the modified E6 and E7, separated by a furin cleavage site (**Figure 1A**).

To confirm that the mRNA can be translated into the theoretical fusion protein, we transfected HEK293T cells with 0.5 µg mRNA, collected whole cell lysates at different time points post-transfection, and detected protein expression below 34 KDa *via* Western Blot with both anti-E6 and anti-E7 antibodies (**Figure 1B**). As expected, the protein expression was transient, it peaked between 24- and 48-hours post-transfection and slowly decayed, with minimal levels detected at day 8 post-transfection (**Figure 1B**). This suggests that the expression and subsequent presentation of the antigens encoded by our mRNA vaccine could be well sustained. We also transfected HEK293T cells with different amounts of mRNA, collected whole cell lysates at 24 hours post-transfection to run Western Blots, and found that the E6-E7 fusion protein expression was titer-dependent (**Figure 1C**). We further investigated the post-translational modifications of our mRNA protein, especially after observing a double band in the Western Blot detecting the E6-E7 fusion protein. NetNGlyc 1.0 predicted the Asparagine at position 195 (NDS consensus sequence) to be N-glycosylated. Hence, we used two approaches, a treatment with tunicamycin and a PNGase glycosidase, to inhibit glycosylation and to cleave glycans in the target protein produced by HeLa cells, respectively. The presence of a single band in correspondence of the E6-E7 fusion protein in the Western Blot (**Figure 1D**) proved that our prediction was correct and confirmed the identity of our target protein.

### 3.2 HPV16 mRNA vaccine induces CTL -mediated immunogenicity

**Figure 2:**
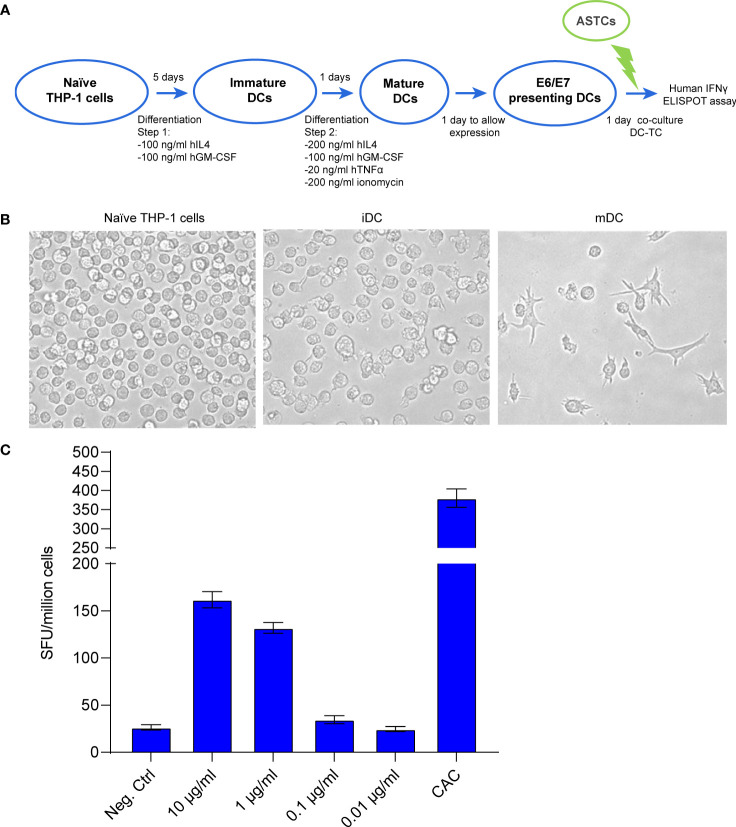
**(A)** Schematic workflow for THP-1 cell differentiation, followed by the coculture of differentiated THP-1 cells with E7 (11-19) antigen specific T cells (ASTCs). DC: dendritic cells (putative) obtained from differentiated THP-1 cells. **(B)** Morphology changes THP-1 cells undergo while differentiating from naïve to immature and mature dendritic cells (iDC, mDC). **(C)** ELISpot assay quantifying the IFNγ positive spot forming units (SFU) derived from the E7(11-19) ASTCs reacting to antigen presentation by THP-1-derived DCs transfected with different concentrations of mRNA.

Towards the goal to prove the functionality for our mRNA-encoded protein, we developed an *in vitro* system to co-culture mRNA-transfected dendritic cells (DC) acting as antigen presenting cells with antigen-specific T cells (ASTC) acting as effector cells. THP-1 cells were differentiated into mature DCs (**Figures 2A, B** and **Supplementary Figure 2**) and then transfected with crescent concentrations of mRNA. The ASTCs were derived from a patient positive to HPV16 and previously characterized for reacting to the HLA-A2-presented peptide E7(11-19). An ELISpot assay was used to quantify the IFNγ positive spot forming units (SFU) derived from the E7(11-19) ASTCs reacting to antigen presentation from THP-1-derived DCs. We found that an increasingly strong IFNγ response was achieved at transfection concentrations of 1 and 10 μg/mL (**Figure 2C**), and concluded that DCs can present immunogenic epitopes derived from our mRNA vaccine.

**Figure 3:**
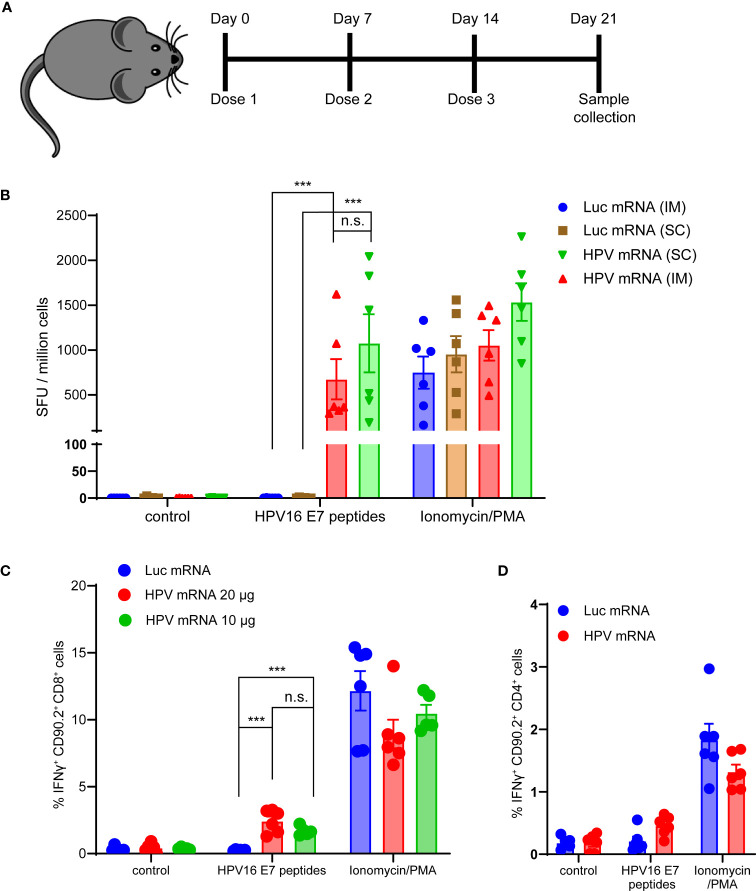
**(A)** Schema of the study design. **(B)** ELISpot analysis of IFNγ positive Spot forming units (SFU) in splenocytes derived from mice vaccinated with 10 μg of MC3-based LNP-formulated mRNA vaccine, given either *via* intramuscular (im) or subcutaneous (sc) injection, and stimulated *ex vivo* with either a vehicle control, 5 μg/mL HPV16 E7 peptides or Ionomycin/PMA control. Data presented as mean ± SEM. Differences between groups were tested using two-way ANOVA test (***, P<0.001; n.s., no significance). **(C)** Flow Cytometry analysis of IFNγ positive Cytotoxic T lymphocytes from splenocytes derived from mice vaccinated with either 10 or 20 μg of MC3-based LNP-formulated mRNA vaccine, or with 20 μg of MC3-based LNP-formulated luciferase mRNA control, and stimulated *ex vivo* with either a vehicle control, 5 μg/mL HPV16 E7 peptides or Ionomycin/PMA control. Data presented as mean ± SEM. Differences between groups were tested using two-way ANOVA test (***, P<0.001; n.s., no significance). **(D)** Flow Cytometry analysis of IFNγ positive Helper T lymphocytes from splenocytes derived from mice vaccinated with either 20 μg of MC3-based LNP-formulated mRNA vaccine, or with 20 μg of MC3-based LNP-formulated luciferase mRNA control, and stimulated *ex vivo* with either a vehicle control, 5 μg/mL HPV16 E7 peptides or Ionomycin/PMA control. Data presented as mean ± SEM. Differences between groups were tested using two-way ANOVA test (n.s., no significance).

Next, we evaluated the T cell-mediated immunogenicity of our mRNA vaccine *in vivo*. Three injections of the mRNA vaccine formulated in MC3-based LNPs were administered in C57BL/6 mice. Seven days after the last dose, mice were sacrificed, spleens were harvested and E6 and E7 antigen specific IFNγ production was measured in splenocytes (**Figure 3A** and **Supplementary Figure 3**). For this purpose, peptide libraries covering the full length E6 and E7 proteins were used in bulks to stimulate splenocytes. As expected, the murine immune response against E6 was too weak (data not shown), while a strong specific immune response against E7 was detected in the mRNA vaccinated mice, but not in the control group immunized with a luciferase-encoding mRNA. The strength of the IFNγ response mounted against E7 was comparable to the nonspecific response induced by the Protein Kinase C (PKC) activator and potent tumor promoter phorbol 12-myristate 13-acetate (PMA) stimulation (**Figure 3B**). We did not observe any significant difference in the IFNγ response between the two different routes of administration tested, intramuscular (im) and subcutaneous (sc) (**Figure 3B**), and we decided to proceed with the clinically relevant im route in the following studies. Additionally, we scrutinized which T cell population was mostly accountable for the IFNγ response induced by the vaccine. We detected a significantly higher percentage of IFNγ positive Cytotoxic T lymphocytes (CTL) in mice vaccinated with either 10 or 20 μg of HPV16 mRNA vaccine than in mice immunized with a Luciferase mRNA (**Figure 3C**). No meaningful increase in CD4+ Helper T cells was observed in the group of animals vaccinated with the HPV16 mRNA (**Figure 3D**).

Finally, we asked whether any humoral response was at play in our immunized mice, that could complement the vaccine-induced adaptive immunity. Low titers of anti-E7 antibodies were detected in the serum of mice dosed with 3 weekly i.m. injections of the vaccine mRNA, and no dose-response dependency was observed (**Supplementary Figure 4**). Taken together, these data show that the immunogenicity of our MC3- based LNP-formulated HPV16 mRNA vaccine is mainly mediated by CD8+ T cells.

### 3.3 HPV16 mRNA vaccine effectively inhibits tumor growth and generates anti-tumoral memory

**Figure 4:**
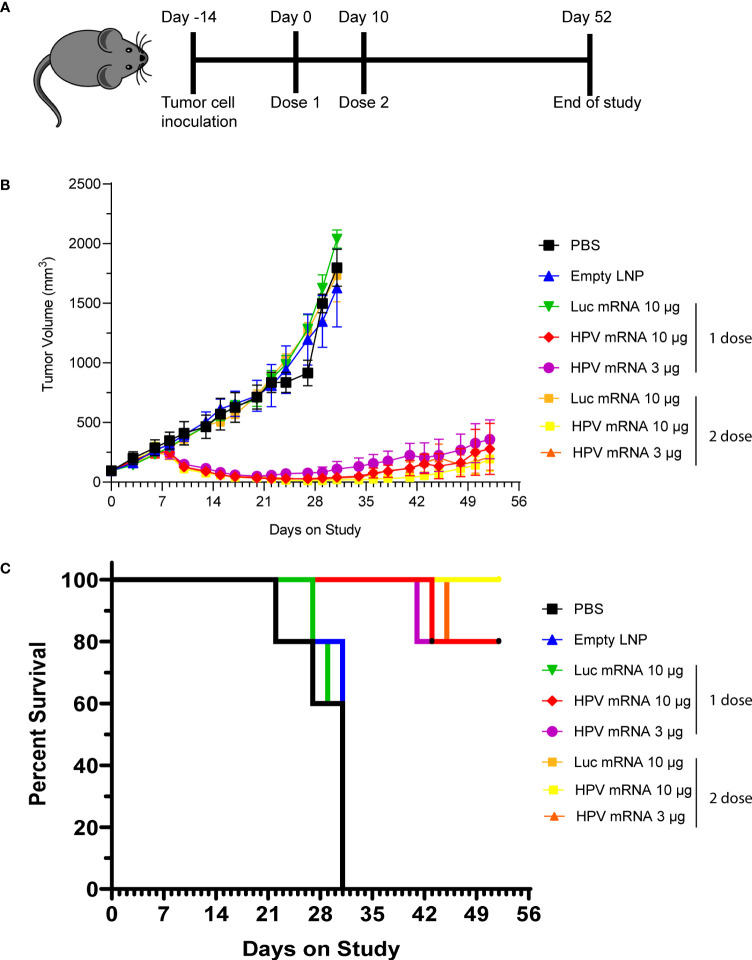
**(A)** Schema of study design, with data presented in **(B, C)**. **(B)** Tumor growth curve of C3.43 tumor bearing mice vaccinated with either a single dose or 2 doses (10 days apart) of HPV vaccine mRNA or Luciferase control mRNA. N=5 mice per group. Data presented as mean ± SEM. **(C)** Survival rates of C3.43 tumor bearing mice vaccinated with either a single dose or 2 doses (10 days apart) of HPV vaccine mRNA or Luciferase control mRNA. N=5 mice per group. Data presented as Kaplan-Meier curves.

We next asked whether the induction of cellular immunity from the mRNA vaccination could result in a durable antitumor effect that can prevent relapse. Hence, we performed the next rounds of experiments by immunizing mice bearing C3.43 tumors. The C3.43 tumor cell line is sub-cloned from C3 cells generated from C57BL/6 mouse embryo cells transformed with an activated-ras oncogene and the complete HPV16 genome (15). As it expresses HPV16 E6 and E7 early proteins, this aggressive tumor cell line is often used for testing the efficacy of immunotherapies aiming at inducing adaptive immunity against HPV16-associated lesions in mouse (16). For our purpose, groups of 5 mice were instilled subcutaneously (s.c.) with C3.43 cells, and subsequently injected with either a single dose or prime-boost doses (10 days apart) of HPV16 mRNA vaccine, once masses reached the 100 mm^3^ volume (**Figure 4A**). Two different doses (3 and 10 μg) were evaluated. Regardless of the regimen, all groups receiving the HPV16 mRNA vaccine exhibited a sharp tumor regression after the first week of treatment, as well as a strong tumor growth inhibition compared to the PBS, empty LNP and Luciferase mRNA control groups (**Figure 4B** and **Table 1**) throughout the entire duration of the study. A dose-dependent improvement in the tumor volumes at day 31 and in the ratio of complete responders at day 52 (**Table 1**) could be noted in the animals injected with 3 μg versus 10 μg of vaccine. All vaccinated mice benefited from an increase in lifespan, and survival was particularly improved (up to 100%) in the group primed and boosted with 10 μg of the HPV16 mRNA vaccine (**Figure 4C**). Furthermore, we selected the prime-boost regimen with the lowest dose of vaccine (3 µg) to profile the ratio of CD4 and CD8 T cells infiltrating the tumor (**Supplementary Figure 5A**). Tumor masses with volume >120 mm^3^ were harvested and the presence of T Helper (CD4+) and Cytotoxic T (CD8+) cells in the single tumors was quantified *via* flow cytometry. The cellular immune response within the tumor milieu appeared strongly skewed towards the CD8+ branch (**Supplementary Figure 5B**). As C3.43 tumors contain the entire HPV genome, we also synthetized mRNA for other HPV16 early proteins (E1, E2, E5) and ran further immunization campaigns to investigate whether an anti-tumor immune response could be primed against other antigens expressed in C3.43 cells. No evidence of tumor inhibition was recorded in any of these animals (**Supplementary Figure 6**).

**Table 1:** The tumor volumes at day 31 and the ratio of complete responders at day 52 presented in Table 1 are relative to the study described in **Figure 4**.

Treatment	N	Tumor Volume (mm^3^) at day31	Complete Response Rate at Day 52
PBS	5	1496.03 ± 78.62	0/5
empty LNP	5	1345.98 ± 216.05	0/5
Luc mRNA_10 μg (1 dose)	5	1624.89 ± 113.18	0/5
HPV mRNA_10 μg (1 dose)	5	34.20 ± 17.73	2/5
HPV mRNA_3 μg (1 dose)	5	82.32 ± 43.80	1/5
Luc mRNA_10 μg (2 dose)	5	1507.73 ± 152.09	0/5
HPV mRNA_10 μg (2 dose)	5	12.29 ± 8.27	2/5
HPV mRNA_3 μg (2 doses)	5	22.76 ± 15.88	1/5

N= number of animals per group.

**Figure 5:**
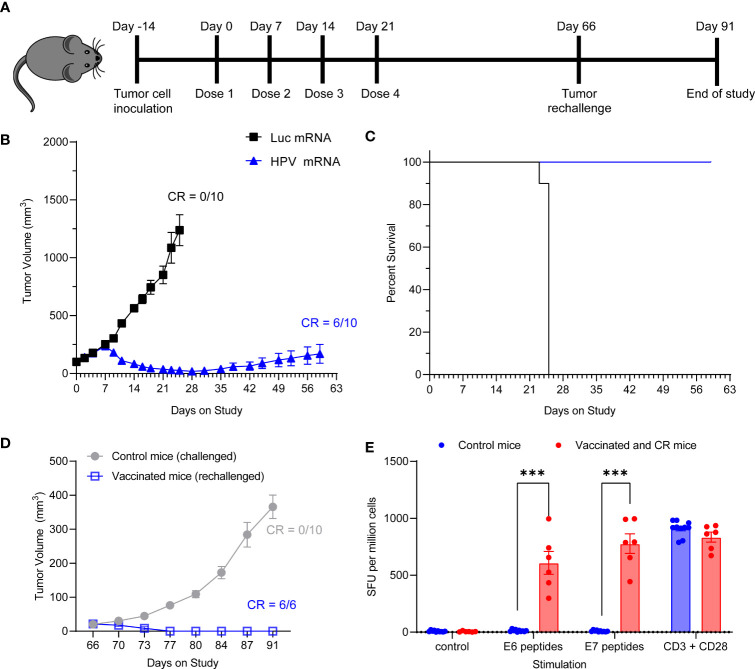
**(A)** Schema of tumor study. **(B)** Tumor growth curve of C3.43 tumor bearing mice vaccinated with four weekly doses of 10 µg HPV vaccine mRNA or Luciferase control mRNA. N=10 mice per group. Data presented as mean ± SEM. CR: complete responders. **(C)** Survival rates of C3.43 tumor bearing mice vaccinated with four weekly doses of 10 μg HPV vaccine mRNA or Luciferase control mRNA. N=10 mice per group. Data presented as Kaplan-Meier curves. **(D)** Tumor growth curve of C3.43 challenged (10 control/never vaccinated mice) and C3.43 rechallenged (the 6 mice who received the vaccine and completely responded by day 60) animals. Data presented as mean ± SEM. CR: complete responders. **(E)** ELISpot analysis of IFNγ positive Spot forming units (SFU) in splenocytes derived from C3.43 tumor bearing- control mice (10 animals, never vaccinated, collected on Day91), -vaccinated and CR mice after rechallenge (6 animals, collected on Day91),and stimulated *ex vivo* with either a vehicle control, 5 μg/mL HPV16 E7 peptides, 5 μg/mL HPV16 E6 peptides or T-activator CD3/CD28 control. Data presented as mean ± SEM. Differences between groups were tested using two-way ANOVA test (***, P<0.001).

We next assessed whether the HPV16 mRNA vaccine could establish protective immune memory. We provided 4 weekly injections of 10 μg HPV16 vaccine to C3.43 tumor bearing mice with established 100 mm^3^ masses (**Figure 5A**). All control mice treated with Luciferase mRNA experienced rapid tumor growth and had to be sacrificed before Day 28, which is shortly after the last control mRNA injection, based on the humane endpoints established by IACUC (**Figure 5B**). Immunization with HPV16 mRNA was associated with a rapid tumor regression after the first week from study enrollment, 100% survival and 60% complete remission by day 59 (**Figure 5B, C** and **Table 2**). All vaccinated mice with a complete anti-tumoral response were then rechallenged with another injection of C3.43 cells and monitored for the remaining time of the study. No mass growth was observed in these mice (**Figure 5D**), indicating that immune memory had been formed in response to the antigens. We went on harvesting the animal spleens at the end of the study to evaluate the IFNγ response of effector T cells upon re-exposure to the antigens. Unvaccinated control mice challenged with C3.43 tumors did not develop any E6 and/or E7 antigen-specific T cells. Vaccinated mice that had a complete tumor regression after the first C3.43 challenge and remained tumor-free after the second C3.43 challenge generated numerous IFNγ-positive T cells in response to stimulation with both E6 and E7 peptide libraries (**Figure 5E**). These findings proved that our HPV16 mRNA vaccine induces growth inhibition and regression of HPV+ tumors and establishes protective immune memory.

**Table 2:** The tumor volumes at day 25 and the ratio of complete responders at day 59 presented in Table 2 are relative to the study described in **Figure 5**.

Treatment	N	Tumor Volume (mm^3^) at Day 25	Complete Response Rate at Day 59
Luc mRNA_10 μg	10	1238.15 ± 133.60	0/10
HPV mRNA_10 μg	10	26.06 ± 10.43	6/10

N= number of animals per group.

## 4 Discussion

We have developed a novel HPV16 mRNA-based vaccine that can elicit an antigen-specific adaptive immune response in a preclinical model of HPV16-associated lesion. This vaccine can be administered intramuscularly and is effective in single and prime-boost dose mode. It can be formulated in LNPs containing a clinically advanced ionizable lipid, MC3, shown to be safe in humans after repeateD Systemsic administrations (17) and proven seroconversion-effective in preclinical and clinical studies on influenza where intramuscular doses of modified mRNA vaccine were tested (18).

HPV prevalence in women is approximately 10% worldwide, with a spike of 22% in Africa, while a broader prevalence range of 1-73% is found in men (19). HPV16, the most prevalent HPV subtype, is detected in the majority of cervical lesions progressing to cancer (3). Cervical lesions can progress through different stages according to dysplasia severity and invasiveness, and are often grouped under the two categories of low- and high-grade squamous intraepithelial lesions (LSIL and HSIL, respectively). HSILs have the lowest rate of spontaneous regression, and if left untreated, may progress to carcinoma (20, 21). Their adequate treatment entails the excision of the entire lesion and transformation zone by loop electrosurgical excision procedure (LEEP) or cold-knife conization, which can result in increased risk of preterm birth and pregnancy loss (22). Thus, a non-invasive therapeutic approach against the HPV16-associated cervical disease is a high unmet medical need. FDA has only approved prophylactic vaccines against HPV16 and other high-risk serotypes, and such vaccines are unable to induce a T-cell mediated immune response against existing precancerous lesions; hence, they are not administered to people with established infections. Moreover, due to the recent approval and use of these prophylactic vaccines (Gardasil^®^ approved by FDA in 2006), to the often-low titers of neutralizing antibodies they induce especially in adult women, and to the suboptimal vaccination rates worldwide (23), HPV infections and development of HPV-associated cancers remains a public health concern and therapeutic vaccines are in need.

HPV infection is usually productive in LSILs, while HSILs are characterized by no virus production, high expression of viral oncoproteins E6 and E7 and integration of the viral DNA into the host DNA (24). The two early proteins E6 and E7 are viral proteins known to interfere with the activity of critical tumor suppressor genes and to mediate evasion of the infected epithelial cells from physiological mechanisms controlling their proliferation (25). E6 degrades p53 through ubiquitination with the help of E6AP (E6-associated protein), thus preventing activation of target proteins p21, Bax and Bak involved in cell cycle arrest and apoptosis; E7 interacts with pRb and targets its ubiquitination, leading to the release of E2F transcription factors, which transcribe cyclin E, cyclin A and p16INK4A, an inhibitor of CDK4/6, forcing the cells through premature S-phase entry. As the biology and pathological involvement of E6 and E7 has been studied for decades, most of the therapeutic vaccines under development are targeting those 2 early proteins.

The Inovio’s DNA-based therapeutic vaccine VGX-3100 targeting E6 and E7 has been recently tested in prospective, randomized, double-blind, placebo-controlled Phase 3 studies (NCT03721978 and NCT03185013) on adult women with histologically confirmed CIN 2 and 3 associated with HPV16 and HPV18. While the resulting histopathological regression and viral clearance following vaccination with VGX-3100 have not been overwhelmingly successful (26), this is the most clinically advanced asset proving that E6 and E7 can be effective targets for an anti-HPV16 therapeutic vaccine. BioNTech’s BNT113 mRNA vaccine targets these antigens, as well, and a phase II trial (27) is ongoing to investigate safety and therapeutic effect in patients with HPV16-positive head and neck cancer. BTN113 preclinical data in mouse showed that E7 immunogenicity can drive growth inhibition of HPV16-positive tumors, though several intravenous injections and a high dose of lipoplex nanoparticle-formulated mRNA were needed to establish a survival advantage in the vaccinated mice implanted with the aggressive C3 tumor cells (28). Recently, an LNP-formulated self-amplifying (sa) RNA containing E7 has been shown to control HPV-associated tumors in mice, too (29). This interesting new modality allows to lower the RNA dose in the vaccine, with positive repercussions on drug manufacturing, but the safety profile of saRNAs remains obscure. Our and others’ studies (28, 29) have the limitation of testing HPV vaccines in naïve C57Bl6 mice, which notoriously struggle to mount a strong immune response against the E6 oncoprotein. It should be noted that Inovio’s VGX-3100 and BioNTech BNT113 vaccines have included E6 for clinical translational purposes, and we are accumulating preliminary data in bigger animal species vaccinated with our E6/E7 mRNA highlighting the immunogenicity of E6 (data not shown). Nevertheless, we have shown that C57Bl6 complete responders rechallenged with the C3.43 tumor were able to mount a vaccine-induced immune response against E6, which suggests that E6 immunogenicity could offer an extra level of protection from recurrency.

Our mRNA has several advantages over other RNA-based HPV16-vaccines: 1) accommodates the two antigens E6 and E7 in a single polypeptide, thus co-administration of two drug products is not required, 2) uses an intramuscular route of injection, thus making patients willing and compliant to receive 1 or more doses of the vaccine, 3) is preclinically effective at single and low dose of 3 μg, without being self-amplifying.

Several vaccines targeting E6 and E7 have been developed in the recent years (9). Nucleic-acid-based vaccines offer several advantages over traditional protein-based vaccines, such as the *in-situ* expression of the antigens and the more natural presentation to the immune system. DNA-based vaccines are usually administered at high doses and *via* electroporation to insure efficient delivery to the nucleus (30). Vaccines based on mRNA do not require electrical impulse device to push the genetic message into the cytosol, and theoretically, they do not integrate into the genome (31). Also, the immunostimulatory effect of their LNP-based formulation and their own nucleotide chemistry can be tuned to act as de facto vaccine adjuvant (32). Other pros of the mRNA modality are the relatively low production costs and the quick manufacturing turnaround, which has contributed to the success of Spikevax^®^ and Comirnaty^®^, two mRNA vaccines that induce protective immunity against the SARS-CoV-2 Spike protein. The ability of mRNA to theoretically encode for any protein and peptide makes mRNA synthesis platforms very flexible, too, to the point that personalized vaccines are no longer a mirage. One of the current limitations of our and others’ HPV vaccines is the restriction to single genotype specific antigens (HPV16’s E6 and E7), as well as the absence of proven overlapping efficacy with other high-risk HPV genotypes, that could strengthen and broaden the immune response against HPV lesions. The intent of the current version of our E6- and E7-targeting vaccine is to treat patients suffering from HPV16-associated lesions. In our studies, we have observed an IFNγ response in patient-derived T cells reacting to putative dendritic cells presenting the vaccine protein. This invigorates our confidence in the translation potential of our vaccine. Another key finding was the reshaping of the C3.43 tumor microenvironment towards a cytotoxic signature. The activation of Cytotoxic T lymphocytes, but not Helper T cells, following antigen stimulation was confirmed by the immunogenicity studies in spleens from vaccinated mice. Based on the recorded humoral response, a meager antibody-dependent cellular cytotoxicity (ADCC) contributing to the elimination of C3.43 tumor cells cannot be excluded. However, the reported humoral immune response evoked by our vaccine will likely have no translational impact in the clinic, in the form of ADCC, as E6 and E7 are expressed intracellularly within the transformed epithelium of the cervix. Finally, the immune memory elicited by our vaccine, alone or in concert with pre-existing tumor reactivities against C3.43 tumor antigens, allowed tumor rejection in 100% of the complete responders without the need for a boosting dose, suggesting that a long-term protection against rechallenge or relapse was induced. Nevertheless, the absence of tumor inhibition observed in C3.43 tumor-bearing mice vaccinated with E1, E2 and E5 suggests that a specific vaccine-induced immune response against E6 and E7 is protective and prevalent over other potential non-vaccine associated immune responses. In conclusion, our mRNA-based therapeutic vaccine targeting the HPV16 oncogenic proteins E6 and E7 holds the promise to become a candidate for future evaluation in clinical trials, and additional pharmacology and toxicology studies in bigger animal species shall help shed some light into its clinical translational potential. If successful in patients, our vaccine could represent a less invasive and risky alternative to surgery for women with HSIL in their reproductive years.

## Data availability statement

The raw data supporting the conclusions of this article will be made available by the authors, without undue reservation.

## Ethics statement

The animal study was reviewed and approved by Biocytogen IACUC Committee.

## Author contributions

KZ, OY, NB and TM contributed to conception and design of the study. LB, DC, DW, XZ, XC and LF contributed to molecule design and preparation. KZ and OY performed the statistical analysis. KZ and TM wrote the manuscript. NB, DW and DC wrote sections of the manuscript. XH and TM provided approval for publication of the content. All authors contributed to the article and approved the submitted version.
