# Supplementary material for: HPV16 E6/E7 -based mRNA vaccine is therapeutic in mice bearing aggressive HPV-positive lesions
Source: Front Immunol. 2023 Jul 12;14:1213285. doi: 10.3389/fimmu.2023.1213285 (PMC10368880; doi:10.3389/fimmu.2023.1213285)
Supplement: Supplementary file 1 [file DataSheet_1.docx]

Supplementary Material

HPV16 E6/E7 -based mRNA vaccine is therapeutic in mice bearing aggressive HPV-positive lesions.

Kun Zhou, Olga Yuzhakov, Nouredine Behloul, Dehua Wang, Lakshmi Bhagat, Dafeng Chu, Xinyue Zhang, Xinwei Cheng, Lusheng Fan, Xinyu Huang, Teodelinda Mirabella^*^

*** Correspondence:** [teodelinda.mirabella@luye.com](mailto:teodelinda.mirabella@luye.com)

# Supplementary Figures and Tables

## Supplementary Figure 1


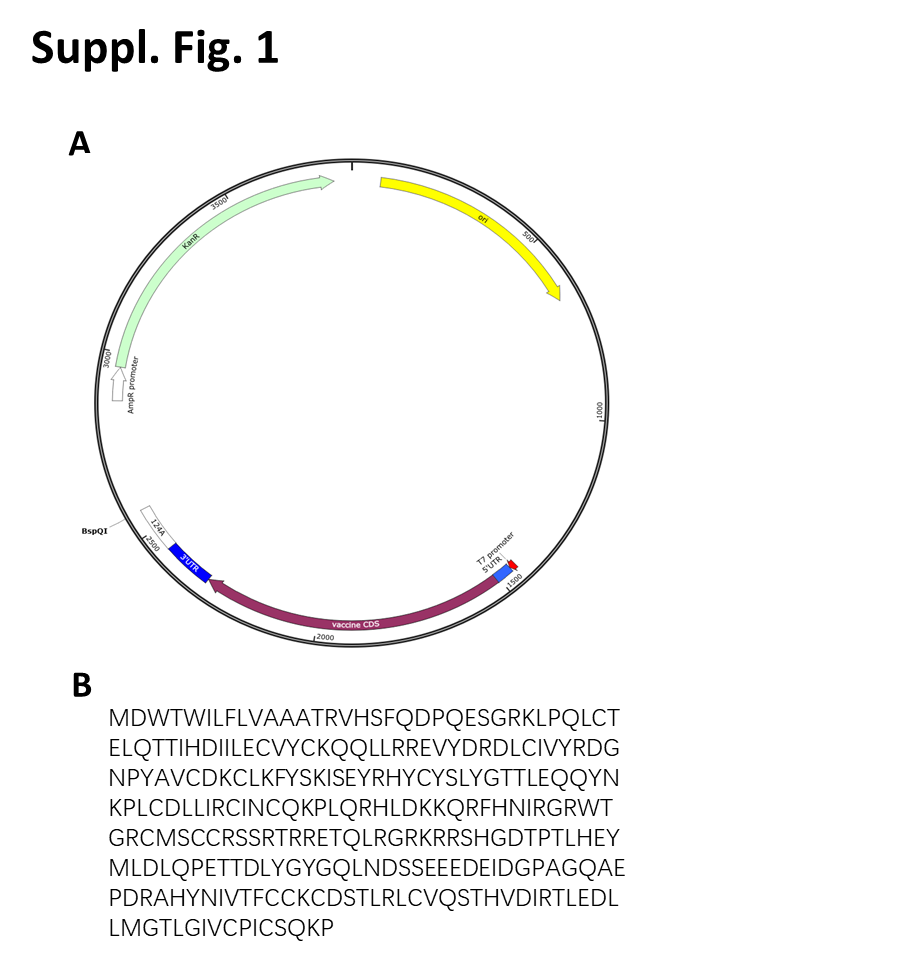


**Supplementary Figure 1.** (A) Plasmid map (SnapGene). (B) Protein sequence (264 aa) encoded by the vaccine mRNA.

## Supplementary Figure 2


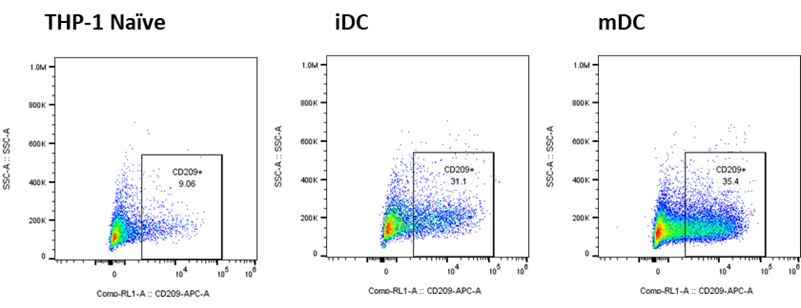


**Supplementary Figure 2.** Flow cytometry to detect differential expression of cluster differentiation markers CD209 in THP-1 cells undergoing differentiation from naïve to immature dendritic cells (iDC) to mature dendritic cells (mDC).

## Supplementary Figure 3


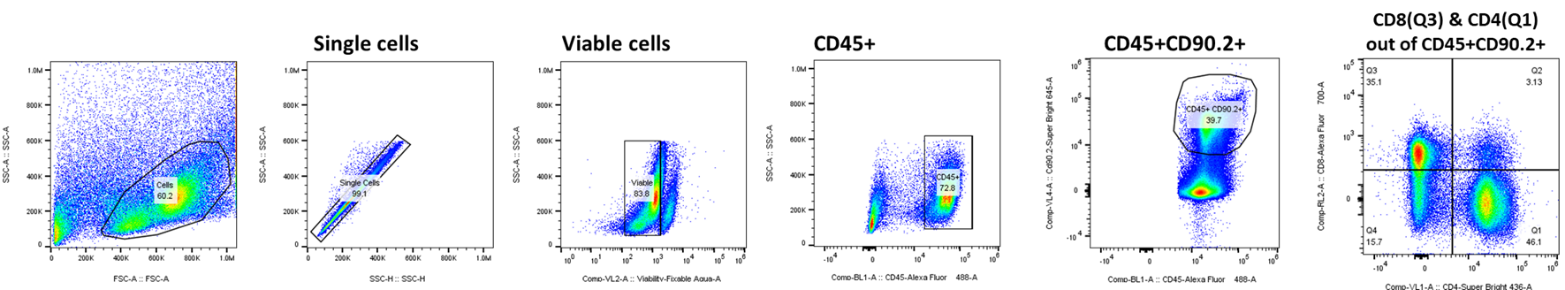


**Supplementary Figure 3.** Flow cytometry gating strategy to selectively analyze CD4 and CD8 positive splenocytes: Single cells> Viable Cells> CD45+ cells > CD90.2+ cells > CD8+ and CD4+ populations.

## Supplementary Figure 4


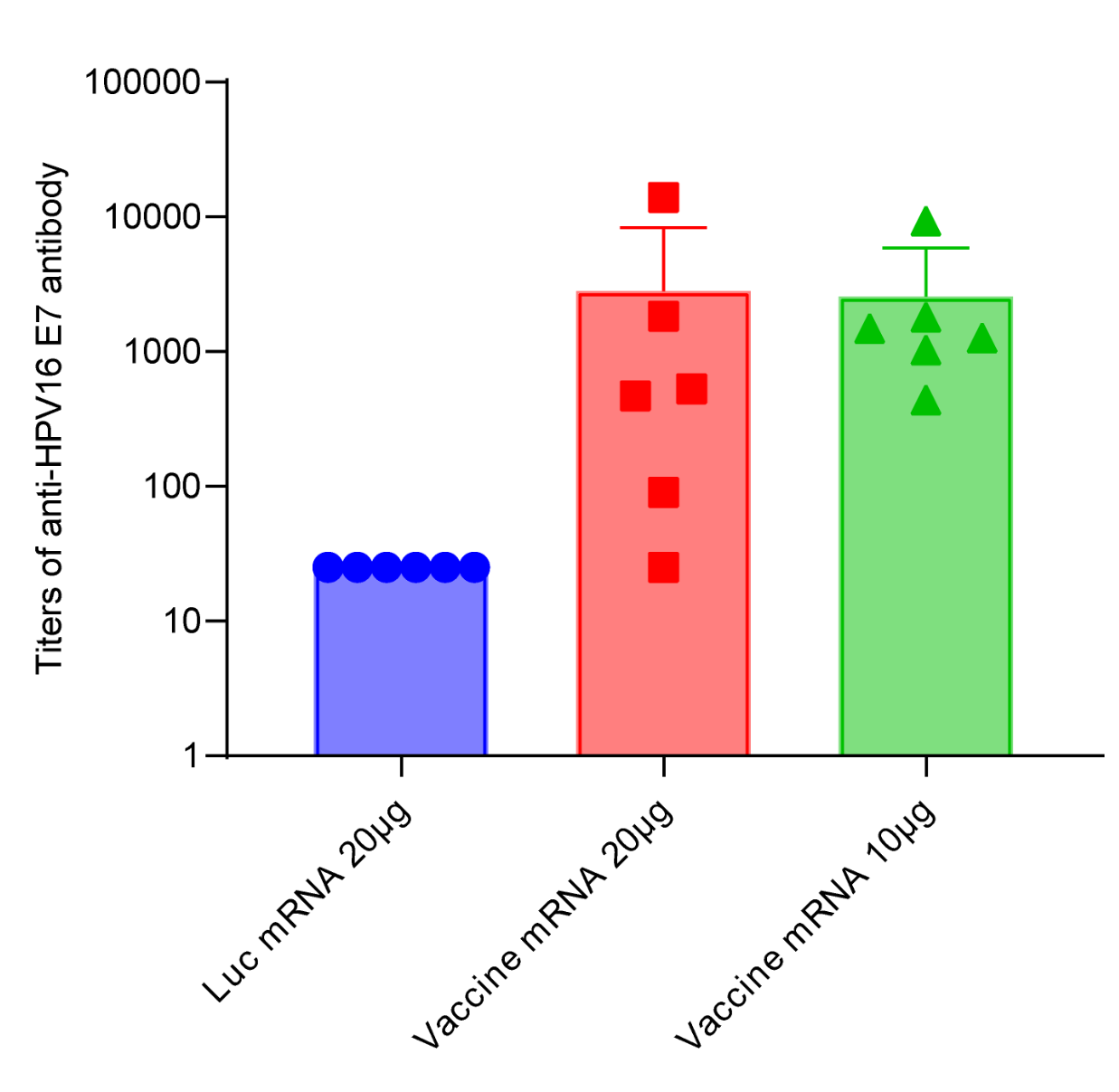


**Supplementary Figure 4.** C57BL6 mice were vaccinated with 3 doses of MC3-based LNP-formulated mRNA (Day 0, 7, 14) and sacrificed one week after the last dose (Day 21) for serum collection. Levels of anti-HPV16 E7 antibodies (IgG) were measured in the serum of the 3 groups (mice injected with 20 µg luciferase mRNA, or 10 µg vaccine, or 20 µg vaccine). Data presented as mean ± SEM.

## Supplementary Figure 5


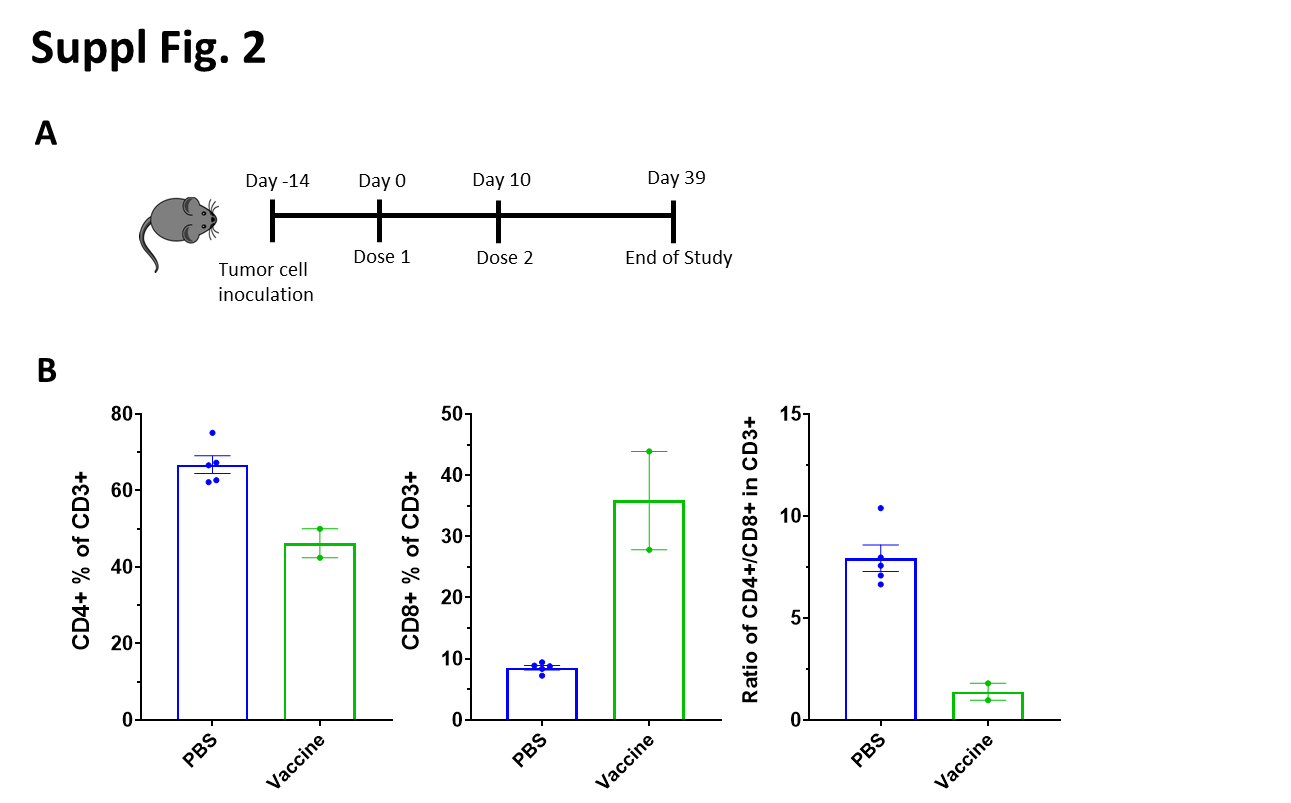


**Supplementary Figure 5.** (A) Schema of study design: C3.43 tumor-bearing mice (N = 5/group) were injected with two doses of 3 μg of mRNA vaccine formulated in MC3-based LNP. Age-matched mice bearing C3.43 tumors (N = 5/group) were later enrolled in the study and injected with PBS. (B) Immune profiling on tumor masses with volume > 120 mm^3^ were recovered at Day 39 for subsequent analysis of percentage of cell populations (CD4 versus CD8 T cells) in the single tumors (N = 5 tumors collected in the PBS group, N = 2 tumors collected in the 3 μg HPV mRNA vaccine group). Data presented as mean ± SEM.

## Supplementary Figure 6


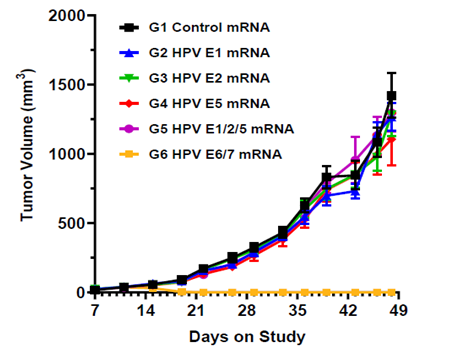


**Supplementary Figure 6.** Seven days after cell inoculation, C3.43 tumor-bearing C57BL6 mice were vaccinated with 3 weekly doses of 10 µg MC3-based LNP-formulated mRNA (Day 0, 7, 14). Several mRNA sequences (with EE > 90%) were tested with N = 10 mice per group: Luciferase (control), E1, E2, E5, a combination of E1-E2-E5, and our candidate vaccine (E6/E7). Mice were monitored for 49 days, with no evidence of antitumoral activity in any of the groups, except in the arm immunized with our candidate vaccine. Data presented as mean ± SEM.
